# Optimization of time frame binning for FDOPA uptake quantification in glioma

**DOI:** 10.1371/journal.pone.0232141

**Published:** 2020-04-22

**Authors:** Antoine Girard, Hervé Saint-Jalmes, Nibras Chaboub, Pierre-Jean Le Reste, Alice Metais, Anne Devillers, Florence Le Jeune, Xavier Palard-Novello

**Affiliations:** 1 Department of Nuclear Medicine, Centre Eugène Marquis, Rennes, France; 2 Department of Nuclear Medicine, Univ Rennes, CLCC Eugène Marquis, Inserm, LTSI – UMR 1099 research unit, Rennes, France; 3 Department of Neurosurgery, CHU Rennes, Rennes, France; 4 Department of Pathology, CHU Rennes, Rennes, France; 5 Univ Rennes, Department of Nuclear Medicine, CLCC Eugène Marquis, “Behavior and Basal Ganglia” research unit, France; Universidad de Navarra, SPAIN

## Abstract

**Introduction:**

3,4-dihydroxy-6-[18F]fluoro-L-phenylalanine (FDOPA) uptake quantification in glioma assessment can be distorted using a non-optimal time frame binning of time-activity curves (TAC). Under-sampling or over-sampling dynamic PET images induces significant variations on kinetic parameters quantification. We aimed to optimize temporal time frame binning for dynamic FDOPA PET imaging.

**Methods:**

Fourteen patients with 33 tumoral TAC with biopsy-proven gliomas were analysed. The mean SUVmax tumor-to-brain ratio (TBRmax) were compared at 20 min and 35 min post-injection (p.i). Five different time frame samplings within 20 min were compared: 11x10sec-6x15sec-5x20sec-3x300sec; 8x15sec– 2x30sec– 2x60sec– 3x300sec; 6x20sec– 8x60sec– 2x300sec; 10x30sec– 3x300sec and 4x45sec– 3x90sec– 5x150sec. The reversible single-tissue compartment model with blood volume parameter (VB) was selected using the Akaike information criterion. K1 values extracted from 1024 noisy simulated TAC using Monte Carlo method from the 5 different time samplings were compared to a target K1 value as the objective, which is the average of the K1 values extracted from the 33 lesions using an imaging-derived input function for each patient.

**Results:**

The mean TBRmax was significantly higher at 20 min p.i. than at 35 min p.i (respectively 1.4 +/- 0.8 and 1.2 +/- 0.6; p <0.001). The target K1 value was 0.161 mL/ccm/min. The 8x15sec– 2x30sec– 2x60sec– 3x300sec time sampling was the optimal time frame binning. K1 values extracted using this optimal time frame binning were significantly different with K1 values extracted from the other time frame samplings, except with K1 values obtained using the 11x10sec– 6x15sec –5x20sec-3x300sec time frame binning.

**Conclusions:**

This optimal sampling schedule design (8x15sec– 2x30sec– 2x60sec– 3x300sec) could be used to minimize bias in quantification of FDOPA uptake in glioma using kinetic analysis.

## Introduction

Gliomas are the second most common primary brain tumor in adults [1]. 3,4-dihydroxy-6-[18F]fluoro-L-phenylalanine (FDOPA) positron emission tomography / computed tomography (PET/CT) is being increasingly used for non-invasive glioma assessment [2–4]. FDOPA is an amino-acid analogue and is used to assess primary brain tumor cell growth [5]. FDOPA PET/CT offers the advantage of detecting both high- and low-grade glioma because FDOPA uptake does not depend on a blood–brain barrier disruption [6, 7]. As recently recommended in the EANM/EANO/RANO practice guidelines/SNMMI procedure standards for imaging of gliomas using PET, the imaging protocol for FDOPA PET/CT consists of a 10–20 min static image acquisition obtained 10–30 min after injection. For routine clinical interpretation, semiquantitative measures of tumor activity uptake values are calculated [8]. However, kinetic parameters obtained through dynamic acquisition might provide further details about tumor characterization [5, 9]. For instance, information regarding tumor aggressiveness from FDOPA PET/CT could have utility in guiding biopsy [10], and potentially improve patient management with dose-escalation using intensity-modulated radiotherapy in patients with glioma [10, 11]. Kinetic analysis mandates time frame binning chosen before reconstruction of dynamic PET images. To the best of our knowledge, no recommendations are available regarding FDOPA PET/CT time frame binning for kinetic analysis in glioma. Up until now, different time frame samplings were used in publications studying glioma uptake quantification using full kinetic analysis for [5, 12–14]. However, we recently showed that a slight difference of temporal sampling induces bias in 18F-Choline uptake quantification in prostate cancer [15]. In this latter study, initial time frame longer than 5 s but also faster than 5 s were not optimal for quantification. The aim of this study was to define an optimal time frame binning protocol for dynamic FDOPA PET imaging.

## Methods

### Patients

Sixteen patients with diffuse glioma were prospectively included in the “GLIROPA” clinical trial (NCT03525080). All gliomas were newly diagnosed and selected for resective surgery. Fourteen patients were analysed because the dynamic acquisition was unsuccessful for 2 patients. There were 9 men and 5 women, with a median age of 40 years (range 23–66). Each patient gave written informed consent prior to inclusion. This study has been performed in accordance with the Declaration of Helsinki, approved by an independent national research ethics committee (Comité de Protection des Personnes Ile de France 1 2018-ND27-cat.2).

### PET/CT imaging protocol

The patients were required to fast at least 4 h before undergoing the imaging protocol. Each patient underwent a CT scan without contrast agent injection, followed by a 40-min PET acquisition using list-mode acquisition with a single field of view centered on the brain (Siemens Biograph mCT, Knoxville, TN). At the start of the PET scan, 2 MBq/kg of FDOPA was administered intravenously, without carbidopa premedication. PET data were reconstructed using Time of Flight (TOF) 3D ordered-subsets expectation maximization iterative algorithm (8 iterations, 21 subsets) with corrections (attenuation, dead time, randoms, scatter and decay) and 4 mm kernel convolution filter. The Point Spread Function reconstruction method was not used as recently recommended in the EANM/EANO/RANO practice guidelines/SNMMI procedure standards for imaging of gliomas using PET [8]. Voxel size was 1x1x2 mm^3^. For each patient, in order to determine the FDOPA bolus arrival time, PET data were reconstructed into 20 frames of 3 seconds (lower bound of time bin reconstruction available on the system).

### Timing of acquisition

A 20-min and 35-min static images from the bolus arrival time were reconstructed. A tumor and a contralateral cortex reference volume-of-interest (VOI) of 1 cm^3^ were generated by a nuclear medicine physician with the Syngo.via software (Siemens) on the 20-min static reconstruction and projected onto the 35-min static reconstruction. Tumor VOIs were drawn based on the MRI-guided brain biopsies (1 cm^3^). The MRI was performed within a median time of 3 days after FDOPA PET/CT and surgery within a median time of 15 days after FDOPA PET/CT. One, two or three biopsies were performed for each patient during surgery. A freehand VOI was drawn using a registration between the MRI used for the MRI-guided brain biopsies and the FDOPA PET/CT with the Syngo.via software. For each voxel, the standardized uptake value (SUV) was calculated calculated using the following formula: SUV = tissue radioactivity concentration /[injected dose /patient weight]. The mean TBR_max_ (tumor SUV_max_/contralateral cortex reference SUV_max_) were compared at 20 min and 35 min post-injection (p.i) using the Wilcoxon signed-rank test for paired samples (IBM SPSS Statistics 25 (SPSS Ltd.). Two-sided values of p < 0.05 were considered significant.

### Time sampling

**Fig 1:**
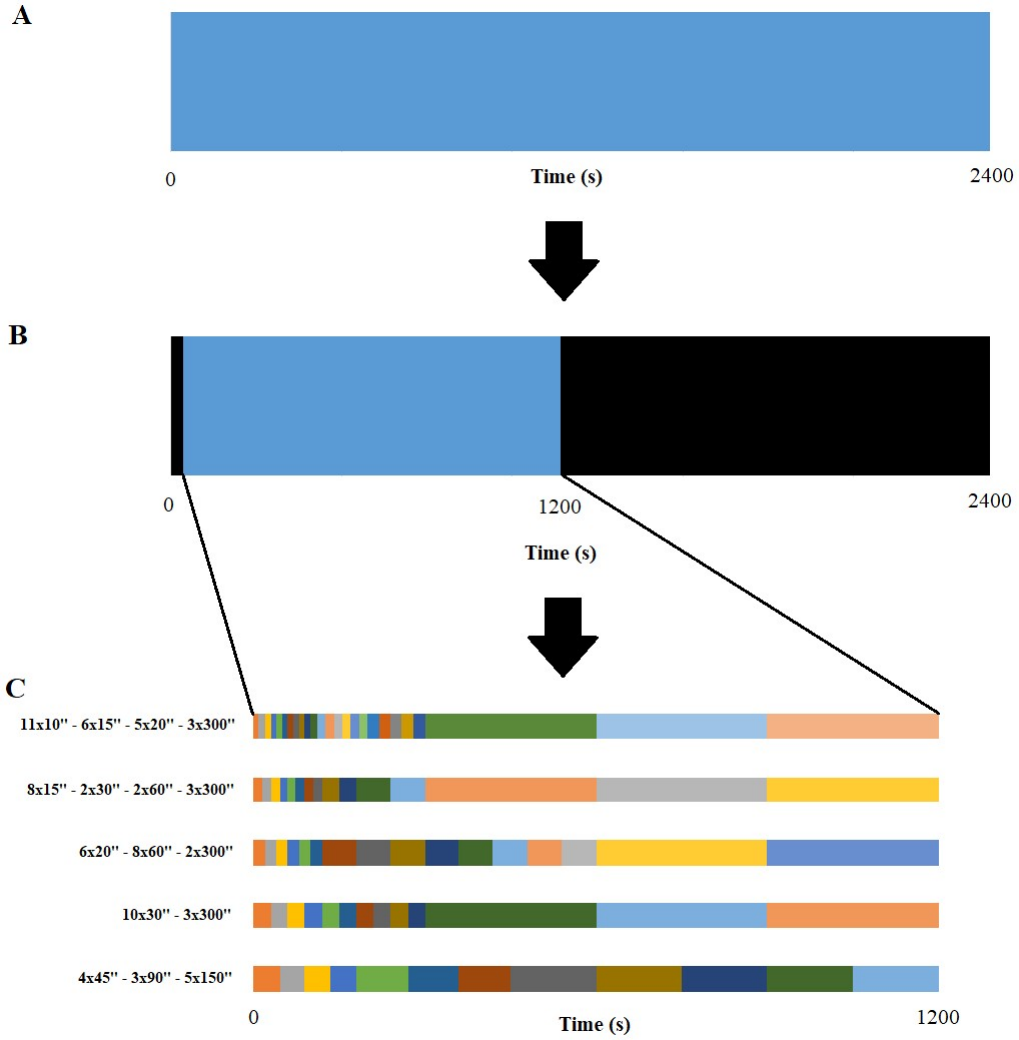
List-mode PET data were recorded during 2400 seconds (**A**). The first seconds without any count were excluded and only 20 minutes from the FDOPA bolus arrival time were selected (**B**). Then, PET data were reconstructed into the 5 different time samplings (**C**).

Five different time samplings with a total study duration of 20 minutes were defined for comparison. Two time samplings were based on previous studies: 8x15sec- 2x30sec - 2x60sec– 3x300sec [5, 12] and 6x20sec- 8x60sec- 2x300sec [13]. In addition, 3 time samplings with different initial frame durations were chosen: 11x10sec- 6x15sec- 5x20sec- 3x300sec; 10x30sec- 3x300sec and 4x45sec- 3x90sec- 5x100sec. 70 reconstructions were performed using list-mode acquisitions (14 patients multiplied by 5 different time samplings from the bolus arrival time (Fig 1)). Tumoral and arterial time-activity curves (TAC) were generated. The tumoral VOI of 1 cm^3^ drawn on the 20-min static reconstruction was projected onto each frame of the 5 different time samplings. On the early PET image with the maximum blood pool activity, a VOI was manually drawn within the middle cerebral artery to estimate an imaging-derived input function (IDIF). For each patient, FDOPA plasma input function was obtained after corrections for metabolites and hematocrit. IDIF was fitted to the measured fractions of metabolites taken from the publication of Huang et al. [16].

### Kinetic model selection

In pharmacokinetic modeling, tracer kinetics are assumed to be separable into compartments with a flux of the tracer from one compartment to another. The flux between compartments can be physical (transport across a membrane) or notional (between bound and unbound receptor or chemical transformation in the same physical space). In the current study, the reversible single-tissue compartment model (1T2k+VB) (with K1 = Rate constant from blood to tissue, k2 = rate constant from the tissue compartment to the arterial blood, distribution volume (DV) = K1/k2 and VB = blood volume parameter), the irreversible (2T3k+VB) and reversible (2T4k+VB) two-tissue compartment model (adding a tissue compartment representing the FDOPA pool of the tumor, with k3 = inward and k4 = outward) were tested (PMOD software version 3.8; PMOD Technologies; Zürich, Switzerland). These three compartmental models are the most commonly used for full kinetic analysis of PET tracers in oncology. The model providing the best fits (Levenberg-Marquadt algorithm) to the tumoral TAC with the 5 different time samplings was selected on the basis of the Akaike information criterion (AIC) for small sample sizes [17].

### Optimal time sampling

Monte Carlo simulations were performed in Mathematica (Wolfram Research, Inc., Mathematica, Version 11.1, Champaign, IL (2017)) in order to determine the optimal time binning between the 5 time samplings tested. The mean of the metabolite-corrected arterial TAC from the fastest initial temporal sampling (11x10sec-6x15sec-5x20sec-3x300sec) extracted from the patients with interpolation to 1-second frames was used for the modeled arterial TAC (C_IDIF_(t)). This modeled arterial TAC was applied for every investigated time sampling. The modeled tumoral TAC C(t) was obtained as follows:

$$\text{C}\left(\text{t}\right)=\text{VB}\;{\text{C}}_{\text{IDIF}}\left(\text{t}\right)+\left(1-\text{VB}\right)\text{K}1\;{\text{e}}^{-\text{k}2\text{t}}⊗{\text{C}}_{\text{IDIF}}\left(\text{t}\right).$$

K1, K2 and VB were average values extracted for the 33 lesions using the 5 different time samplings according to the selected model.

For each time sampling, 1024 realizations of independent distributed Poisson noise (Added noise) were added to the modeled TAC as follows:

AddedNoise=c(RandomInteger[PoissonDistribution[C(t)]]−C(t))/Sqrt(dt),

where c is the scaling factor and dt is the frame duration.

Each realization was fitted to the model providing an estimation of the kinetic parameters. The mean and standard deviation of the estimated K1 values were computed from all the realizations and compared to a target K1 value as the objective, which is the average of the K1 values extracted from the 33 lesions.

### Clinical validation

Comparison of all of the K1 values extracted from the optimal time sampling with K1 values extracted from the other time samplings was performed using the nonparametric Wilcoxon signed-rank test for paired samples [18] because the data was not normally distributed. Two-sided values of False Discovery Rate adjusted p < 0.05 were considered significant. Statistical analysis was performed using IBM SPSS Statistics 25 (SPSS Ltd.).

### Correlations between the different imaging parameters

IDIF based Logan graphical analysis was also performed. The distribution volume (Vt) was calculated as the slope of the linear part of the Logan analysis. The relationship of the different imaging parameters extracted using the optimal time sampling was investigated using Spearman’s correlation coefficient (r). A p value less than 0.05 was considered significant.

## Results

### Patients

**Fig 2:**
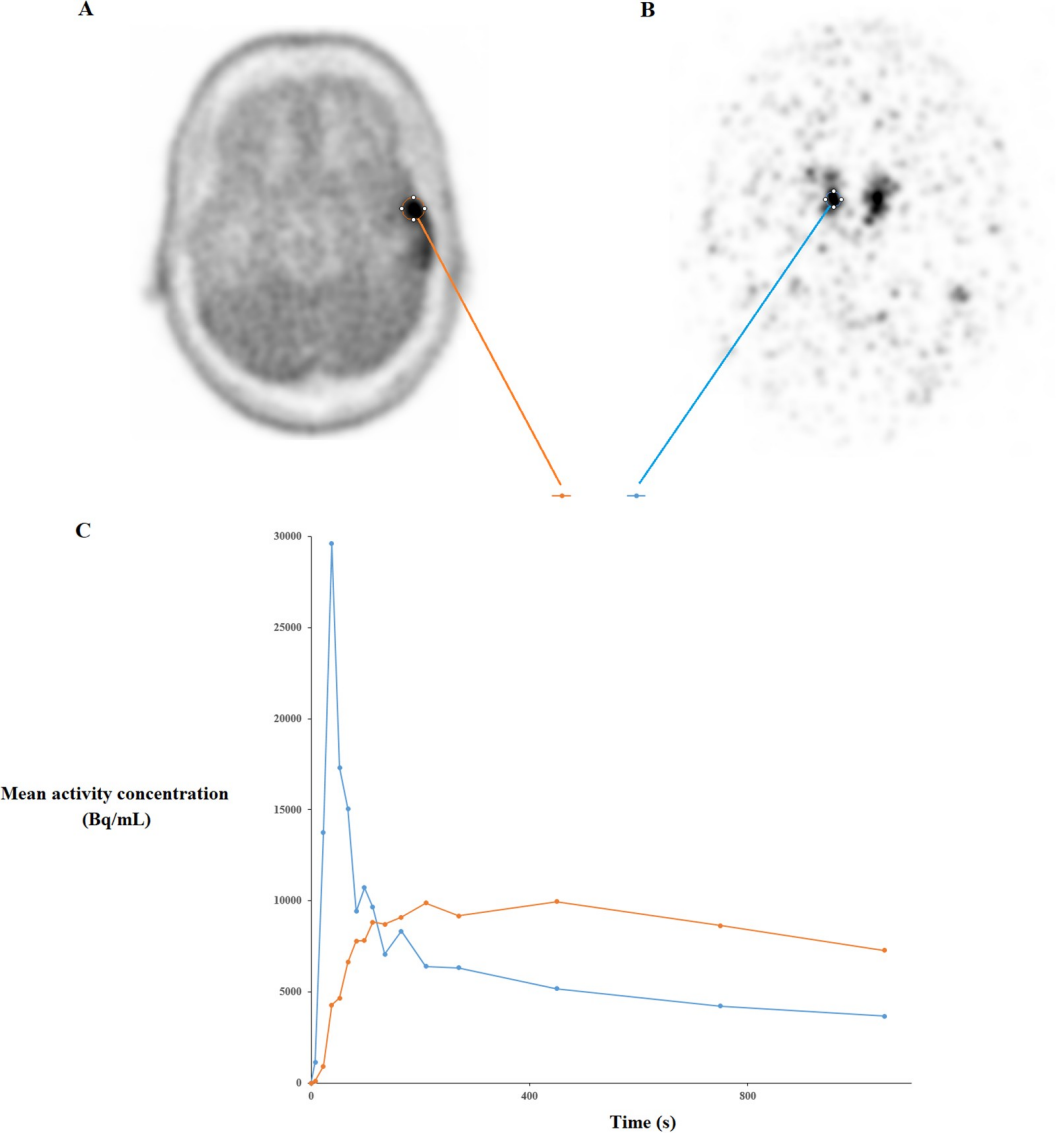
Axial FDOPA PET images show glioma uptake in a 50-year-old man (A) and the injected FDOPA bolus (176 MBq) on the right middle cerebral artery (B) with arterial and glioma time-activity curves (C).

Thirty-three biopsies were done. The distribution of the fourteen cases on the basis of the 2016 World Health Organization histopathologic classification was as follows: 6 patients with astrocytoma, 2 patients with oligodendroglioma, and 6 patients with glioblastoma. Typical TAC in a 50 years old man are shown in Fig 2.

### Timing of acquisition

**Fig 3:**
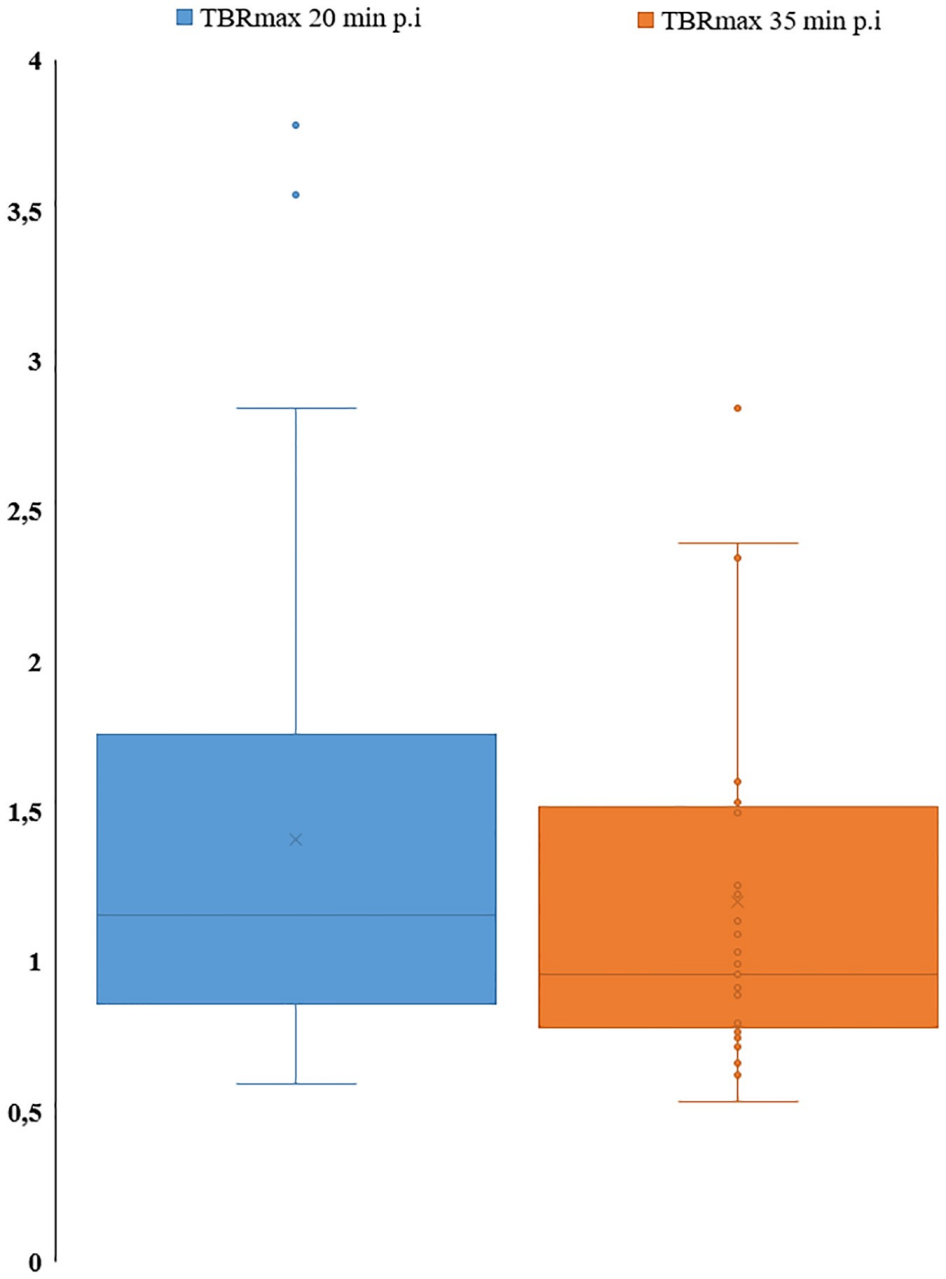
Box plots for TBRmax at 20 min p.i and 35 min p.i.

The mean TBR_max_ was significantly higher at 20 min p.i. than at 35 min p.i (respectively 1.4 +/- 0.8 and 1.2 +/- 0.6; p <0.001) (Fig 3).

### Kinetic model selection

AIC results indicated that the 1T2k+VB model produced the best fits (preferred model in 102 (62%) of the 165 tumoral TAC from all of the time samplings). The mean K1 value according to the 1T2k+VB model for all of the lesions from all of the time samplings was 0.161 mL/ccm/min. The mean k2 was 0.087 min^-1^. The mean VB was 8.4%.

### Optimal time sampling

The average K1 value of the 1024 simulations obtained by the 8x15sec– 2x30sec– 2x60sec– 3x300sec time sampling was the closest value to the target K1 value (0.161 mL/ccm/min) (Table 1). The K1 values extracted from the simulated TAC with the latter time sampling were significantly different with the K1 values extracted using the other time samplings, except with the 11x10sec– 6x15sec–5x20sec– 3x300sec time sampling.

**Table 1:** Comparison of the average K1 values from the 1024 simulations (Monte Carlo) using the 5 different time samplings.

Time sampling	Average K1 value (mL/ccm/min^-^) +/- SD	95% Confidence Interval (mL/ccm/min)	
		Lower bound	Upper bound
11x10sec-6x15sec-5x20sec-3x300sec	0.1621 +/- 0.0146	0.1612	0.1630
**8x15sec– 2x30sec– 2x60sec– 3x300sec**	**0.1604 +/- 0.0128**	**0.1596**	**0.1612**
6x20sec– 8x60sec– 2x300sec	0.1634 +/- 0.0124	0.1627	0.1642
10x30sec– 3x300sec	0.1690 +/- 0.0152	0.1689	0.17
4x45sec– 3x90sec– 5x150sec	0.1745 +/- 0.0131	0.173	0.1753

### Clinical validation

Results showed that the K1 values extracted from the optimal time sampling (8x15sec– 2x30sec– 2x60sec– 3x300sec) for the 33 lesions were significantly different with the K1 values extracted using the other time samplings tested, except for the comparison with the 11x10sec– 6x15sec–5x20sec– 3x300sec time sampling (Table 2).

**Table 2:** Comparison of the K1 values extracted from the optimal time sampling (8x15sec– 2x30sec– 2x60sec– 3x300sec) and K1 values from the other time samplings for the 33 lesions.

Time sampling	Average K1 value (mL/ccm/min) +/- SD	p value
11x10sec-6x15sec-5x20sec-3x300sec	0.214 +/- 0.2	0.178
6x20sec– 8x60sec– 2x300sec	0.143 +/- 0.12	0.003*
10x30sec– 3x300sec	0.151 +/- 0.124	0.042*
4x45sec– 3x90sec– 5x150sec	0.146 +/- 0.130	0.002*

*p values <0.05 = statistically significant

### Correlations between the different parameters

Using the optimal time sampling (8x15sec– 2x30sec– 2x60sec– 3x300sec), the mean DV was 1.68 mL/ccm +/- 0.65 and the mean Vt obtained using the Logan graphical analysis was 1.68 mL/ccm +/- 0.56. Correlation between SUVmax and K1 and between SUVmax and Vt were high (respectively r = 0.88, p < 0.001 and r = 0.77, p < 0.001). All of the correlation coefficients are given in Table 3.

**Table 3:** Spearman’s rank correlation matrix for the imaging parameters.

	**K1**	**k2**	**DV**	**Vb**	**SUVmax**	**TBRmax**	**Vt**
**K1**	1	0.83*	0.65*	0.61*	0.88*	0.90*	0.67*
**k2**		1	0.21	0.48*	0.60*	0.68*	0.29
**DV**			1	0.55*	0.78*	0.73*	0.87*
**Vb**				1	0.60*	0.63*	0.31
**SUVmax**					1	0.96*	0.77*
**TBRmax**						1	0.69*
**Vt**							1

*p values <0.05 = statistically significant

## Discussion

Under-sampling or over-sampling induces significant variations on kinetic parameters quantification [19]. In order to optimize the quantification of dynamic FDOPA uptake, the aim of this study was to define the optimal temporal sampling for FDOPA PET/CT reconstruction protocol in patients with glioma.

Firstly, our results showed that TBRmax was significantly higher at 20 min p.i than at 35 min p.i. Moreover, the TBRmax was always higher at 20 min p.i than at 35 min p.i for all tumors. Several studies have previously explored the evolution of glioma FDOPA uptake over time. Chen et al. showed that the highest tumor FDOPA uptake occurred between 10 min and 30 min after injection [12]. Similarly, in the study published by Schiepers et al., the tumor FDOPA uptake peak activity was reached around 20 min p.i [5]. In two more recent studies, the TACs of tumor FDOPA uptake peaked earlier at 8–10 min p.i [9, 13]. However, the EANM/EANO/RANO practice guidelines/SNMMI procedure standards for imaging of gliomas using PET recently recommended a 10–20 min image acquisition performed 10–30 min p.i [8].

Secondly, among 5 different time frame binning protocols, the results of this study show that the 8x15sec– 2x30sec– 2x60sec– 3x300sec time sampling is optimal. Using full quantification, two studies compared FDOPA influx with tumor grade. On the one hand, Schiepers et al. suggested that newly diagnosed high-grade brain tumors had significantly higher K1 values than K1 values extracted from low-grade brain tumors [5]. On the other hand, Kratochwil et al. found no significant difference of K1 values between high-grade and low-grade brain tumors [13]. A possible explanation of these discordant results could be linked to the dynamic temporal sampling protocol. The protocol was different in the two latter studies. The results of our study show that K1 values extracted using full kinetic analysis depend on the time frame binning protocol. The optimization of the temporal resolution during kinetic acquisition not only concerns FDOPA but also the quantitative analysis of other PET radiopharmaceuticals [15, 19–20]. We previously demonstrated that a better estimation of ^18^F-Choline uptake quantification is obtained using an initial time frame of 5s in prostate cancer assessment using the same PET system [15], shorter than the optimal initial frame of 15 s for FDOPA quantification in glioma assessment in this study. A lower activity concentration of FDOPA in glioma than activity concentration of ^18^F-Choline in prostate cancer might be the reason. Indeed, emission events rate in PET modality can be described as a Poisson distribution. Poor counting statistics need longer frames. Regarding FDOPA PET/CT dynamic imaging protocol, to the best of our knowledge, no guidelines are available and no previous research has investigated the optimization of the time frame binning. However, list-mode data cannot be stored on a clinical picture archiving and communication system. Temporal sampling has to be defined to store the dynamic PET data.

Thirdly, based on the Akaike criterion, the reversible single-tissue compartment with blood volume fraction was the preferred kinetic model to describe FDOPA uptake in glioma. To the best of our knowledge, only two studies evaluated compartment modeling for FDOPA uptake quantification in glioma based on dynamic PET scans [5, 9]. Schiepers et al. demonstrated that the error estimates are significantly smaller for the two-tissue compartment model than for the one single-tissue compartment model [5]. In our study, the PET study duration for the kinetic analysis was 20 min whereas it was 75 min in the Schiepers et al study. This difference of duration could explain why the selected compartment model was different between the latter study and our study. Indeed, Kratochwil et al. suggested that K1 was predominant in the FDOPA uptake in the first minutes post-injection [13]. Nioche et al. study results confirmed this finding, showing that the FDOPA uptake in glioma extracted using the two-tissue compartment model and the uptake using the single-tissue compartment model were very close with a PET study duration of 45 minutes [9].

Fourthly, this study showed a strong correlation between SUVmax and uptake rate constant as determined either by graphical Logan analysis or pharmacokinetic modeling. A simpler static measure in place of dynamic PET scans for quantifying FDOPA uptake in glioma should be sufficient in clinical practice. However, other studies are needed to confirm these results.

This study has several limitations. First, the number of patients was limited, although the number of samples is relatively large. Second, the input function used for the PET kinetic modeling was not obtained from arterial sampling. However, FDOPA plasma input function was obtained after corrections for metabolites and hematocrit, based on a previous publication data [16]. Recent studies also used an imaging-derived plasma input function for quantifying FDOPA glioma uptake [5, 9, 12–14]. Third, variations in methodological factors such as FDOPA dose, non-TOF PET system, image reconstruction, post-filtering and tracer kinetic modeling could bias K1 estimates. The 8x15sec– 2x30sec– 2x60sec– 3x300sec temporal sampling was found to be optimal with the parameters of a modern PET system. Fourth, only 33 lesions was analysed for the clinical validation. Further studies with larger number of lesions are needed to confirm the results of our study.

## Conclusion

This optimal sampling schedule design (8x15sec– 2x30sec– 2x60sec– 3x300sec) could be used to minimize bias in quantification of FDOPA uptake in glioma using kinetic analysis.
